# EMU: reconfigurable graphical user interfaces for Micro-Manager

**DOI:** 10.1186/s12859-020-03727-8

**Published:** 2020-10-15

**Authors:** Joran Deschamps, Jonas Ries

**Affiliations:** European Molecular Biology Laboratory, Meyerhofstrasse 1, Heidelberg, Germany

**Keywords:** Microscopy, User interface, GUI, Micro-Manager

## Abstract

**Background:**

Advanced light microscopy methods are key to many biological studies. Their ease of use depends, besides experimental aspects, on intuitive graphical user interfaces (GUI). The open-source software Micro-Manager offers a universal GUI for microscope control but requires implementing plugins to further tailor it to specific systems. However, GUIs are often tailored to a single system. Since even similar devices can have different Micro-Manager device properties, such as power percentage versus absolute power, directly transferring a GUI to another instrument usually requires changing the source-code.

**Results:**

We developed Easier Micro-Manager User interface (EMU), a Micro-Manager plugin, to simplify building flexible and reconfigurable GUIs. EMU can be seamlessly used with the Java Swing library to create device-independent GUIs for Micro-Manager. Such GUIs are easily transferred to another microscope thanks to an intuitive configuration menu that includes mapping of the device properties to the GUI functionalities and customization of the graphical elements. We also provide resources such as user and programming guides, a tutorial and code examples.

**Conclusions:**

Micro-Manager users now have a powerful tool to improve the user experience on their instruments. EMU GUIs can be easily configured for new microscopes and shared with other research groups. In the future, newly developed GUIs will be added to EMU to benefit the whole community.

## Background

Light microscopy is an ever-growing field with countless applications in biosciences. A substantial portion of technology developments and cutting-edge research are carried out on custom microscopes because of their high flexibility. Beyond the mechanical and optical requirements of such microscopes, researchers face the challenges of controlling the hardware and presenting the users with an intuitive graphical user interface (GUI). While control software are often developed for a specific instrument, a number of more general platforms are available. These software can be commercially available, such as MetaMorph (Molecular Devices), SlideBook (3i), SciScan (Scientifica) or ScanImage (Vidrio) [1], or shared online by their authors. They span a variety of programming languages, allowing users to work with their preferred tools and facilitating post-processing and analysis. Examples of such microscope control software are found for LabView (National Instruments) [2], Matlab (MathWorks) [3] or Python [4–7]. Another well-established alternative is Micro-Manager (µManager) [8], an open-source software written in C++ and Java, and based on ImageJ [9].

µManager is a ready-to-use platform compatible with a wide range of hardware devices. The support for new devices is community driven and constantly expanding. In addition, µManager features the possibility to run scripts or plugins to perform custom experiments. µManager offers a universal GUI and some customization tools that are easy to set-up and use. However, because they are aimed at covering general needs, they cannot rival with a tailored GUI in terms of user experience. The preferred way to implement a GUI in µManager is by writing a plugin in Java, which is automatically detected at start-up and can be loaded from the main menu. Several dedicated Java libraries exist in order to build a GUI, among which the widely used Swing toolkit [10]. In order to simplify the programming task, most major Java integrated development environments provide graphical tools based on Swing. With only a basic understanding of Swing, developers can assemble complex GUIs by placing (“drag-and-drop”) components on a panel or a frame. In order to control a microscope, the Swing GUIs should be made compatible with µManager plugin system and call its application programming interface (API) to modify the devices’ states whenever users interact with the graphical components.

Yet, similar devices might have different properties, such as absolute laser power versus laser power percentage; as well as different property state values, such as “On” versus “1”. These differences originate from the absence of industry standards for device communication API or simply from distinct inherent working principles. Additionally, device properties are defined in µManager device adapters, which are implemented by the manufacturers or individual researchers, and lack device property standardization as well. Therefore, in order to benefit the community, µManager GUIs should be flexible enough to accommodate these discrepancies without requiring modifying the source code.

Several GUIs have been developed to control complex microscopes with µManager, such as for the Olympus IX83 [11], OpenSPIM [12], OpenSpin [13] and diSPIM [14] microscopes. Because these GUIs perform specialised acquisitions and hardware control, they are meant to work with very specific hardware devices. As a result, any departure from the original devices requires modifying the source-code to achieve compatibility. Regrettably, this is a common trait of GUIs, which are generally bound to a single system and never benefit other users.

Here, we present a framework, called Easier Micro-Manager User interfaces (EMU), allowing developers to build µManager GUIs without explicit references to the devices or their properties. While EMU GUIs are, as previously, assembled using Swing, they additionally declare settings, properties and parameters. These are automatically aggregated upon starting EMU and can be configured through an intuitive graphical interface. The EMU configuration specifies the mapping between device and GUI properties, as well as the settings’ and parameters’ values. Thus, each EMU GUI is independent from the devices and can be rapidly configured to suit a microscope or accommodate device exchanges. Finally, EMU aims at offering a repository of already existing GUIs for the community to use.

## Implementation

EMU is a µManager 2.0.0-gamma plugin. It is written in Java and includes its own plugin system. EMU can be started from the plugin menu of µManager. When starting EMU for the first time, users can choose a GUI among the list of available EMU plugins, before configuring it using the EMU configuration menu. The configuration file is saved locally and is loaded automatically at the next start. In the next sections, we showcase how to implement an EMU plugin with code examples and how to configure the plugin in µManager.

### Implementing an EMU plugin

**Fig. 1:**
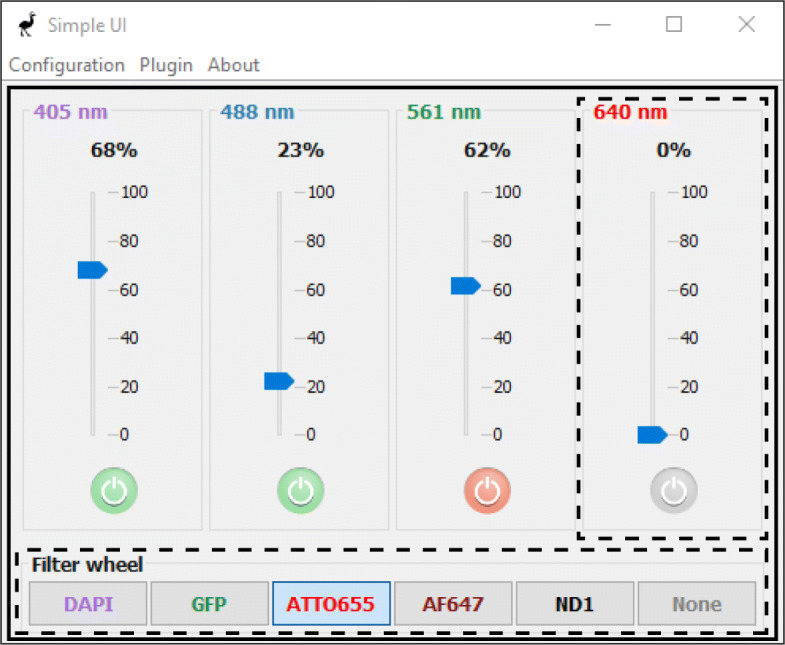
EMU plugin example. SimpleUI is a GUI available by default in EMU. It can control four lasers and an optional filterwheel. The solid box delimits the *ConfigurableMainFrame* instance while the dashed boxes are the two base *ConfigurablePanel* objects of the GUI. All titles and colors are GUI parameters, while the GUI properties are, for each laser, the power percentage and on/off, and the filter wheel position. Additional GUI parameters allow disabling the on/off buttons. Finally, a plugin setting hides or shows the filter wheel panel

The EMU framework is based on the Java Swing toolkit [10]. EMU plugins consist of multiple *ConfigurablePanel* objects arranged within a single *ConfigurableMainFrame* instance, as illustrated in Fig. 1. *ConfigurablePanel* and *ConfigurableMainFrame* are subclasses of the Swing classes *JPanel* and *JFrame*, respectively.

A *ConfigurablePanel* is a unit of device control. In the following code snippet, we create a subclass of *ConfigurablePanel* similar to the laser panel of Fig. 1, with a slider to set the laser power and a toggle button to turn it on or off. We also create a border, with title, around the graphical components. Since *ConfigurablePanel* is a subclass of *JPanel*, the laying out of components is performed using Swing:


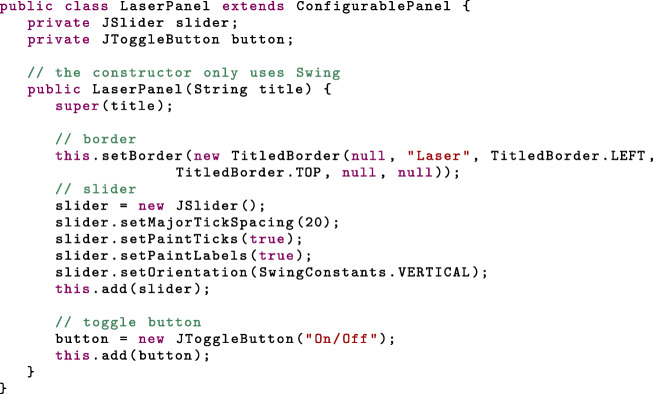


Several abstract methods from *ConfigurablePanel* must additionally be implemented (see Additional file 1). Three of these methods concern the GUI properties: *initilizeProperties*, *addComponentListeners* and *propertyhasChanged*.

**Fig. 2:**
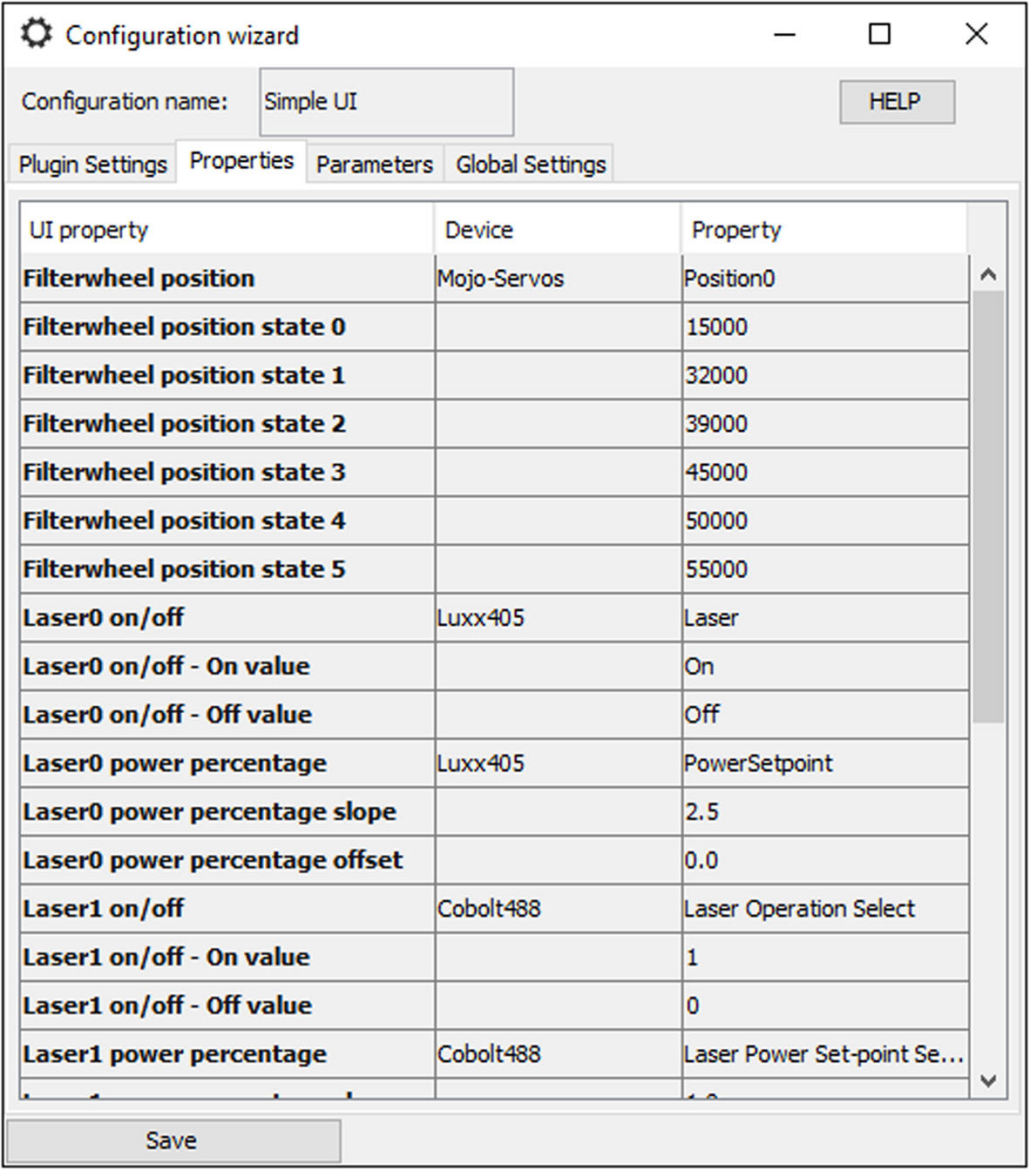
Configuring GUI properties. In the “Properties” tab of the EMU configuration menu, users can map device properties to GUI properties using drop-down lists. Additionally, some GUI properties have state values than need to be specified. Here shown for SimpleUI

In *initilizeProperties*, we need to declare the GUI properties. Since we want to control two device properties, laser power and laser on/off, we have to declare two GUI properties. Different *UIProperty* subclasses exist (see Table 1 and Additional file 2), and the choice of the class depends mainly on the graphical components to which it is linked. For instance, the slider allows setting a value between 0 and 100 by default. As we do not know whether the device property ultimately linked to the slider will be a power percentage or an absolute power, we should declare a *RescaledUIProperty*. In the EMU configuration menu, this type of property allows users to set scaling factors mapping the slider value to a suitable range with respects to the device property (see Fig. 2). Likewise, the laser operation is controlled by a toggle button. Therefore we choose a property with only two states (*TwoStateUIProperty*). The on and off states are set in the EMU configuration menu as well. Declaration of the GUI properties is done as follows:

**Table 1:** *UIProperty* class and child classes. A *UIProperty* object only changes the state of a device property within the µManager device property limits or allowed values. *UIProperty* child classes have additional constraints, as specified in the second column. In the EMU configuration menu, these constraints lead to additional fields as indicated in the third column

Class	Note	in EMU configuration menu
*UIProperty*	General GUI property	device and property drop-down lists
*SingleStateUIProperty*	Accepts a single-state	+ field for the state value
*TwoStateUIProperty*	Accepts an *On* and an *Off* state	+ fields for the *On* and *Off* values
*MultiStateUIProperty*	Accepts a fixed number of states	+ field for each state value
*RescaledUIProperty*	Rescales value to slope*v+*offset*	+ fields for the *slope/offset* values


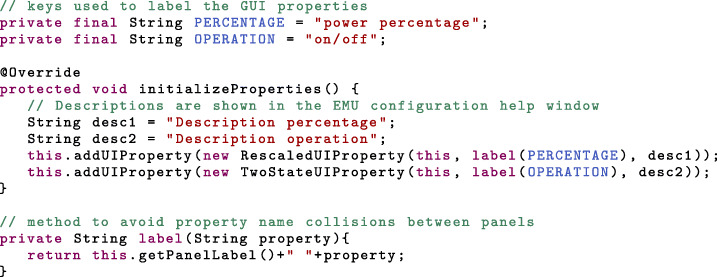


When the user interacts with a graphical component, the state of the corresponding device property should change. In Swing, you can register a listener with a component, the former being called when the component’s state is modified. In a listener implementation, the *ConfigurablePanel* should call *setPropertyValue(String, String)* (see Additional file 1) to update the GUI property given the component’s new state. In turn, the *UIProperty* will change the state of the device property. The listeners can be registered with the components in the constructor or in the *addComponentListeners* method. Alternatively, the EMU *SwingUIListeners* class provides a number of static methods that cover common cases. For instance, here, a convenient way to have the slider and toggle button modify their respective properties is:


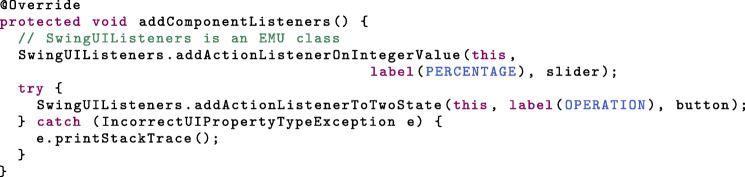


Whenever the state of the graphical components needs to be updated, the *ConfigurablePanel* instances are notified, which triggers the *propertyhasChanged* method. In this method, we need to update the state of the graphical components to reflect the device properties’ values. Here, this means setting the slider value to the (rescaled) laser power, or switching the toggle button to the state of the laser operation. This is done in the following way:


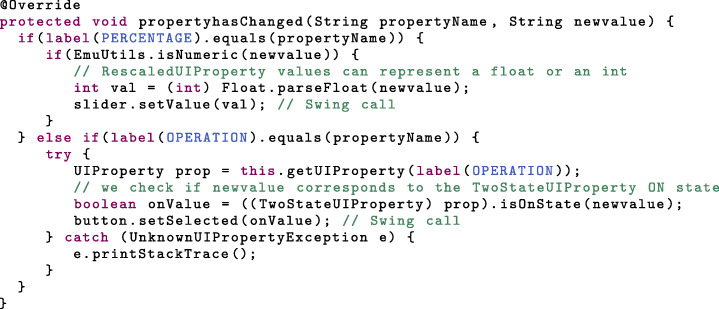


Note that here, we made use of a *TwoStateUIProperty* class method (see Additional file 2).

With these three methods implemented, any numerical device property can be linked to the slider, while any device property can be switched between two states by the toggle button.

Another aspect of EMU panels are the GUI parameters, which are meant to add additional levels of customization to the panel, such as title, colors or button texts. Similarly to the properties, they are declared in *initializeParameters*. Parameters are not modified by the panel and their value only changes at start-up when the configuration is loaded, or when a new configuration is saved, which triggers a call to the *parameterhasChanged* method. Various types of parameters are available in EMU (see Table 2 and Additional file 3).

**Table 2:** *UIParameter* child classes. Each *UIParameter* child class holds a member variable represented by the type indicated in the second column. In the EMU configuration menu, the GUI parameters appear as specified in the third column

Class	Parameter type	in EMU configuration menu
*BoolUIParameter*	Boolean	checkbox
*ColorUIParameter*	java.awt.Color	drop-down list of colors
*ComboUIParameter*	String	drop-down list of Strings
*DoubleUIParameter*	Double	field
*IntegerUIParameter*	Integer	field
*StringUIParameter*	String	field
*UIPropertyParameter*	String	drop-down list of *UIProperty* labels

Since we added a titled border to the panel, we can use a *StringUIParameter* to let users choose the border title:


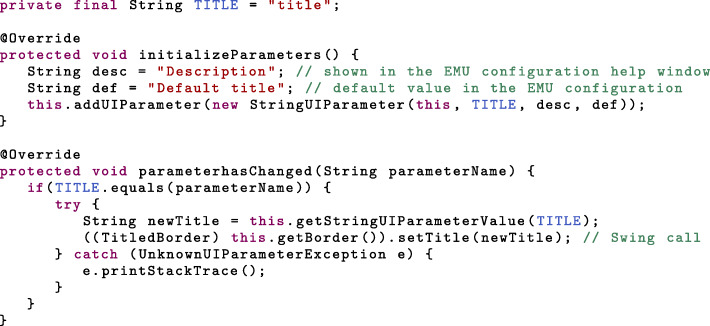


Other mechanisms make EMU panels flexible but are beyond the scope of this section, for instance: internal properties (see Additional file 4), which are values shared between panels; the possibility to map multiple GUI properties to the same device property; or the mapping of a GUI property to a bundle of device properties, known as “configuration group preset” in µManager.

Finally, the *ConfigurablePanel* subclasses are assembled within a single *ConfigurableMainFrame* subclass. The latter declares its own parameters, called settings (see Table 3 and Additional file 5), which are instantiated in *getDefaultPluginSettings*. In the same vein as GUI properties and parameters, their values are defined in the EMU configuration. *ConfigurablePanel* subclass instances should be created in the *initComponents* methods, in order to be able to retrieve the settings’ values at runtime. In the following example, the *ConfigurableMainFrame* subclass uses an *IntSetting* to let users choose the number of lasers in the GUI.

**Table 3:** *Setting* child classes. Each class holds a member variable represented by the type indicated in the second column. All *Setting* child classes appear in the EMU configuration menu as a field, except for the *BoolSetting* settings, which appear as checkboxes

Class	Setting type	in EMU configuration menu
*BoolSetting*	Boolean	checkbox
*DoubleSetting*	Double	field
*IntSetting*	Integer	field
*StringSetting*	String	field


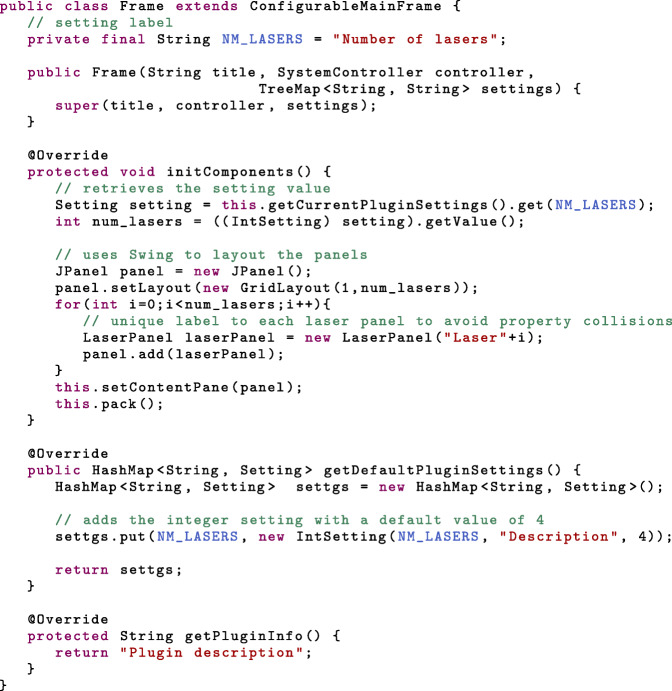


In order to be detected by EMU, one additional class is required (*UIPlugin*) before packaging the GUI into a.jar file. The detailed steps can be found in the EMU guide [15], including the source code for the plugin of Fig. 1. In the next section, we will look at a the configuration of the latter.

### Configuring an EMU plugin

The EMU configuration menu is shown each time a plugin without known calibration is started. Later, it can be accessed by clicking on the menu bar “Configuration” (see Fig. 1), then “Modify configuration”. The EMU configuration menu consists of four tabs: “Plugin Settings”, “Properties”, “Parameters” and “Global Settings”.

In the “Properties” tab (see Fig. 2 for SimpleUI), users can map µManager device properties to GUI properties by first selecting the device in the drop-down list of the second column, then the relevant device property in the third column. Some GUI properties have additional states that need to be configured as well (see Table 1). In particular, this is the case for the GUI properties used in the previous section. States are related to the actual device property values and can be easily inferred from the “device property browser”. The latter is accessible from the “Devices” menu in µManager main window.

**Fig. 3:**
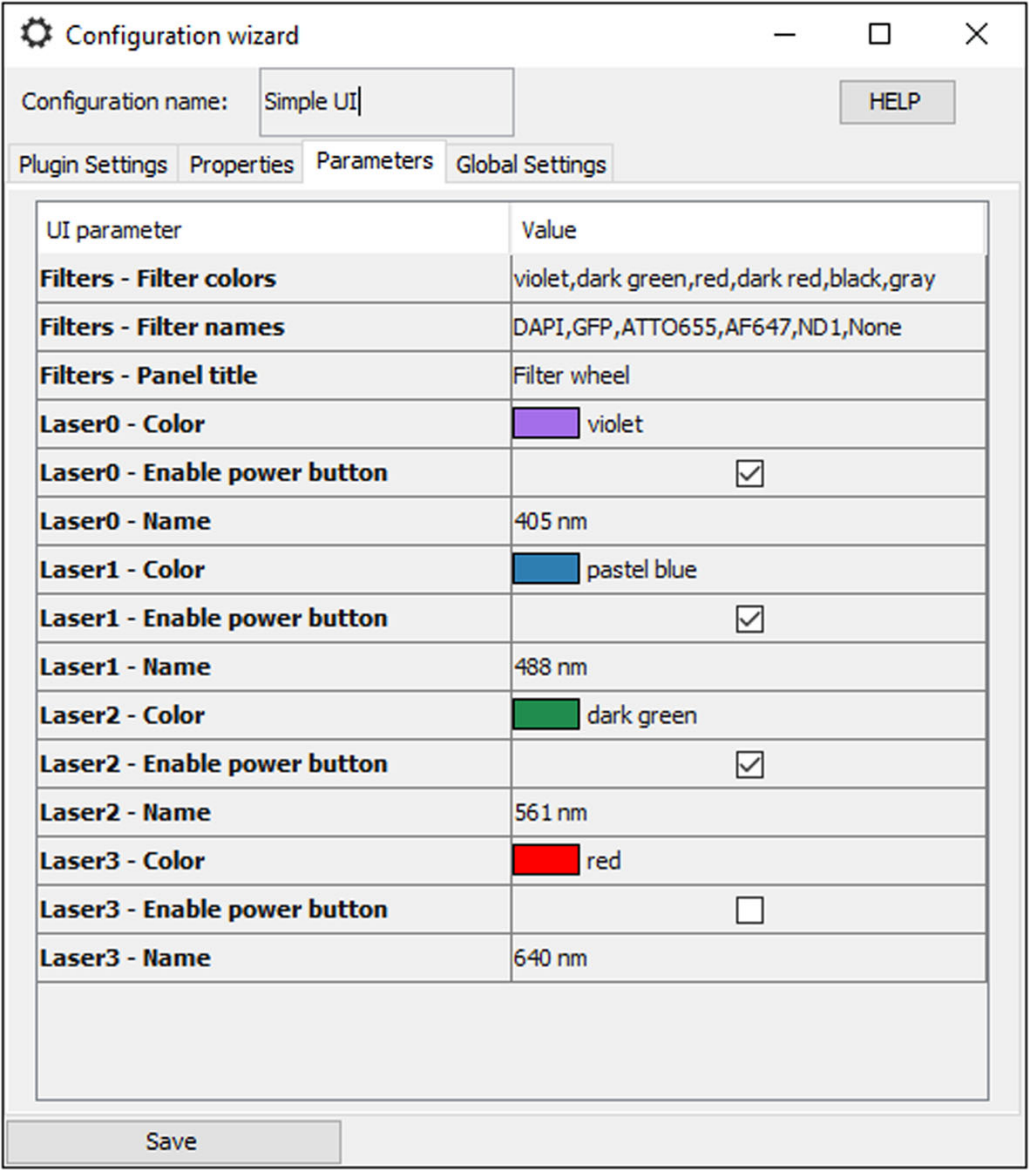
Configuring GUI parameters. In the “Parameters” tab of the EMU configuration menu, users can set the values of the GUI parameters. Here shown for SimpleUI

In the “Parameters” tab (see Fig. 3), each GUI parameter appears as a field, a drop-down list or a checkbox, depending of their type (see Table 2). Finally, the other tabs are the frame settings (“Plugin Settings”) or EMU options (“Global Settings”).

After saving the EMU configuration, a human-readable file is automatically created in a subfolder of the µManager installation folder. Multiple configurations can coexist and be saved in the EMU configuration file. The EMU menu (see the menu bar in Fig. 1) allows users to switch between GUIs or between configurations in a single click. Finally, at each start of EMU, the configuration file is loaded and the last known plugin configured.

### Example cases

To illustrate the flexibility of EMU plugins, we can consider a simple example such as SimpleUI (see Fig. 1). The plugin controls four lasers, allowing users to turn their emission on and off and change their power percentage. Laser names and colors can be set in the EMU configuration menu (see Fig. 3). Several cases can be encountered when working with lasers in µManager: (i) lasers have an operation (on/off) and a power percentage device property, (ii) some lasers have an absolute power instead of a power percentage device property and (iii) some lasers do not have a laser emission device property [16]. In (i), the GUI properties can be mapped to the devices and their relevant device properties. Since the GUI property representing the laser percentage is an instance of *RescaledUIProperty* (see Table 1), users can leave the *slope* and *offset* values (see Fig. 2) equal to the default 1 and 0, respectively. For the lasers in (ii), the laser percentage GUI property is mapped to an absolute power device property. There, the *slope* parameter should be set to *max*/100 (with *max* being the maximum laser power), effectively rescaling the power percentage to the range {0,*max*} of the device property. Finally, for lasers in the case (iii), no laser operation device property exists. The laser operation GUI properties should remain unconfigured. Using the GUI parameters, the on/off buttons can be disabled. In all three cases, the GUI can be configured to reflect an accurate picture of the microscope lasers in spite of their differences.

The previous example plugin is simple. However there is no limit on how complex an EMU plugin can be. We routinely use htSMLM [17] (see Additional file 6), an EMU plugin aimed at controlling a wide-field microscope for localization microscopy [18–20]. htSMLM features four generic lasers, two optional lasers compatible with the iBeamSmart series from Toptica, an axial focusing panel, up to four filter wheels and multiple toggle buttons. It also includes tools to automate activation in localization microscopy and to perform acquisition series (localization microscopy, multi-slice localization, snapshot, time series, z-stack) that take into account the device properties linked to the GUI. htSMLM has been used by scientists with a wide variety of backgrounds and projects, ranging from biology to optics [21–24].

## Discussion

µManager GUIs are too often tailored to a specific system and are not usable for instruments with similar, yet different, devices. Therefore, such GUIs are only shared when the system has the potential to be broadly replicated. Users without programming skills are limited to the µManager main GUI window for device control. While the main GUI covers general need and is thus applicable to most instruments, user experience can be largely improved by using more specialized GUIs in conjunction with the main window. EMU fills this gap by providing device-independent and easily configurable GUIs for µManager.

Since microscopes are composed of a wide variety of devices, the diversity and redundancy of devices prevent automated allocation of the device properties to graphical components without using stringent constraints. In order to give a high degree of flexibility to developers, EMU consists of a set of classes that can be seamlessly inserted in a Swing GUI. The two main components, frames and panels, declare parameters matching the constraints of the graphical components rather than those of the intended devices. Hence, assumptions are limited to whether a certain type of device can be controlled by a specific graphical component. For instance, laser power is a device property that is undoubtedly determined by a number within a range and can therefore be represented graphically by a slider linked to a *RescaledUIProperty*. Similarly, a filter wheel has fixed positions, regardless of whether the actual device positioning is continuous or discrete, and can be paired with a set of buttons linked to a *MultiStateUIProperty*.

The implementation and graphical layout of an EMU GUI is left to developers. Because EMU provides an advanced configuration system and the mechanism of interaction with the device properties, they can focus solely on the GUI design and the choice of properties and parameters fitting the GUI desired functionalities. EMU automatically aggregates the GUI properties and parameters in its configuration menu, which makes the framework particularly beneficial for complex GUIs. Moreover, EMU is compatible with “drag-and-drop” software found in most integrated development environment. Since EMU’s API is designed to be simple and involves only a few additional lines of code, plugins can be built and put to use rapidly.

The goal of EMU is to promote GUIs that are transferable between similar instruments. Indeed, a GUI taking advantage of EMU’s flexibility will be usable on any other microscope consisting of similar devices. Nonetheless, a few limitations can be encountered. If the GUI was designed to work with a device property specific to a single manufacturer, then the corresponding functionality will not be of interest to other users. In such a case, developers should ensure that settings or parameters can be used to simply disable this functionality. As an example, htSMLM was designed to control, among others, iBeamSmart lasers (Toptica). Since these lasers have unconventional properties, such as the so-called fine settings, the corresponding panels can be disabled using parameters. Note that unused properties do not alter the function of the rest of the GUI. Similarly, limitations stemming from the plugin design itself can occur. For instance, a maximum laser power device property expected to set the upper bound of a slider is obviously not compatible with lasers that do not have such a property. Using a parameter to set the maximum value is an easy work-around for developers. Alternatively, the slider could represent a percentage, rather than an absolute value, and be linked to a *RescaledUIProperty*. Finally, unconventional device property implementations can potentially be found in µManager, such as numerical device properties implemented with a string type. These would prevent allocating it to *RescaledUIProperty* GUI properties, which can only be paired with float or integer device properties. Other GUI properties (see Table 1), on the other hand, are compatible with any device property.

EMU includes an intuitive configuration menu allowing users to map their instrument device properties to the GUI properties, as well as setting the various parameters to improve the GUI friendliness and further tailor it to the microscope. Configuring an EMU plugin for an instrument only takes a couple of minutes. In the same way, exchanging devices on the instrument is equally convenient, as users only need to start the EMU configuration menu and change the relevant lines to reflect the presence of the new device. It is worth noting that the EMU configuration is different from the µManager configuration. The latter describes which devices are loaded in µManager and how communication is performed, while the former defines the EMU GUI look and which device properties are linked to the GUI functionalities. EMU plugins are therefore complementary to the µManager main window and an obvious consequence is that only devices compatible with µManager can be used with EMU.

In order to help developers use EMU, we provide online a variety of resources [15], including a user and a programming guide, a step-by-step tutorial on how to create an EMU plugin, as well as multiple code examples exploring all aspects of EMU’s API. EMU is included in the latest µManager distribution and comprises two example plugins.

## Conclusion

µManager is a successful open-source software and is widely used to control custom microscopes. However, its universal interface does not provide a user experience comparable to tailored GUIs. Moreover, most GUIs are developed for a specific system and are never used elsewhere. Here, we presented EMU, a framework that allows developers to build device-independent GUIs that can easily be transferred to another microscope, as well as to other research groups. It also includes an intuitive graphical configuration menu, enabling users to rapidly tailor a GUI to their instrument. We hope that in the future developers will contribute their own GUI to EMU in order to benefit the whole community. The source code can be found on Github following the link in reference [25].

## Availability and requirements

**Project name:** emu

**Project home page:**https://jdeschamps.github.io/EMU-guide

**Operating system(s):** Platform independent

**Programming language:** Java

**Other requirements:** Micro-Manager 2.0.0-gamma

**License:** LGPL-2.1

**Any restrictions to use by non-academics:** None

## Supplementary information

**Additional file 1** UML diagram: *ConfigurableMainFrame* and *ConfigurablePanel*. Unified Modeling Language (UML) diagram of the *ConfigurableMainFrame* and *ConfigurablePanel* classes. The two abstract classes inherit from the Swing classes *JFrame* and *JPanel*, respectively. Additionally, *ConfigurableMainFrame* implements the *ConfigurableFrame* interface. As both classes are abstract, their abstract methods must be implemented by developers when creating the *ConfigurableMainFrame* subclass and *ConfigurablePanel* subclasses. Each class is represented by a box with three compartments: class name, class variables and class methods. Abstract class and interface names are written in italic. Inheritance relationship between two classes is shown as a solid line with an arrow pointing towards the superclass, while implementation of an interface is shown as a dashed line with an arrowhead pointing to the interface class. Aggregation, or class instances being owned by another class, is shown as a solid line with diamond head. The visibility of member variables and methods is indicated by the following signs: **-** (private), **#** (protected) or **+** (public). The diagram shows all private member variables as well as all non-private member methods. Abstract methods are displayed in italic. Variable and method return types, if applicable, are indicated after a colon. The corresponding Javadoc is available in the EMU source-code [25].

**Additional file 2** UML diagram: *UIProperty* and subclasses. UML diagram of the *UIProperty* class and its subclasses. These classes are instantiated in *ConfigurablePanel* subclasses and are ultimately mapped using the EMU configuration to µManager device properties. Descriptions of each class’ specificity can be found in Table 1. Each class is represented by a box with three compartments: class name, class variables and class methods. Inheritance relationship between two classes is shown as a solid line with an arrow pointing towards the superclass. The visibility of member variables and methods is indicated by the following signs: **-** (private), **#** (protected) or **+** (public). The diagram shows all private member variables as well as all non-private member methods. Static methods are underlined. Variable and method return types, if applicable, are indicated after a colon. The corresponding Javadoc is available in the EMU source-code [25].

**Additional file 3** UML diagram: *UIParameter* and subclasses. UML diagram of the abstract *UIParameter* class and its subclasses. These classes are instantiated in *ConfigurablePanel* subclasses and their respective member variable *value_* is set to a user-defined value using the EMU configuration. Each class is represented by a box with three compartments: class name, class variables and class methods. Abstract class names are written in italic. Parameterized class types are shown in a box on the top right corner of each class. Inheritance relationship between two classes is shown as a solid line with an arrow pointing towards the superclass. The visibility of member variables and methods is indicated by the following signs: **-** (private), **#** (protected) or **+** (public). The diagram shows all private member variables as well as all non-private member methods. Abstract methods are displayed in italic. Variable and method return types, if applicable, are indicated after a colon. The corresponding Javadoc is available in the EMU source-code [25].

**Additional file 4** UML diagram: *InternalProperty* and subclasses. UML diagram of the abstract *InternalProperty* class and its subclasses. Internal properties are shared between *ConfigurablePanel* subclasses, provided that they are of the same type and are given the same label. Each class is represented by a box with three compartments: class name, class variables and class methods. Abstract class names are written in italic. Parameterized class types are shown in a box on the top right corner of each class. Inheritance relationship between two classes is shown as a solid line with an arrow pointing towards the superclass. The visibility of member variables and methods is indicated by the following signs: **-** (private), **#** (protected) or **+** (public). The diagram shows all private member variables as well as all non-private member methods. Abstract methods are displayed in italic. Variable and method return types, if applicable, are indicated after a colon. The corresponding Javadoc is available in the EMU source-code [25].

**Additional file 5** UML diagram: *Setting* and subclasses. UML diagram of the abstract *Setting* class and its subclasses. These classes are instantiated in the *ConfigurableMainFrame* subclass and their respective member variable *value_* is set to a user-defined value using the EMU configuration. Each class is represented by a box with three compartments: class name, class variables and class methods. Abstract class names are written in italic. Parameterized class types are shown in a box on the top right corner of each class. Inheritance relationship between two classes is shown as a solid line with an arrow pointing towards the superclass. The visibility of member variables and methods is indicated by the following signs: **-** (private), **#** (protected) or **+** (public). The diagram shows all private member variables as well as all non-private member methods. Abstract methods are displayed in italic. Variable and method return types, if applicable, are indicated after a colon. The corresponding Javadoc is available in the EMU source-code [25].

**Additional file 6** htSMLM plugin. htSMLM is a complex EMU plugin aimed at controlling a wide-field or localization microscope. Besides the controls for multiple lasers, filter wheels and focus, it features tools to perform series of sequential acquisition (e.g. localization microscopy, multi-slice localization, time series or z-stack) and automated laser activation.

## Data Availability

EMU is distributed with Micro-Manager 2.0.0-gamma nightly-builds. The source code, guide, tutorial and examples are available at: https://github.com/jdeschamps/EMU https://jdeschamps.github.io/EMU-guide.

## Abbreviations

API: Application programming interface
EMU: Easier Micro-Manager User interfaces
GUI: graphical user interface
µManager: Micro-Manager
