# Supplementary figures and images for: EMU: reconfigurable graphical user interfaces for Micro-Manager
Source: BMC Bioinformatics. 2020 Oct 15;21:456. doi: 10.1186/s12859-020-03727-8 (PMC7559757; doi:10.1186/s12859-020-03727-8)

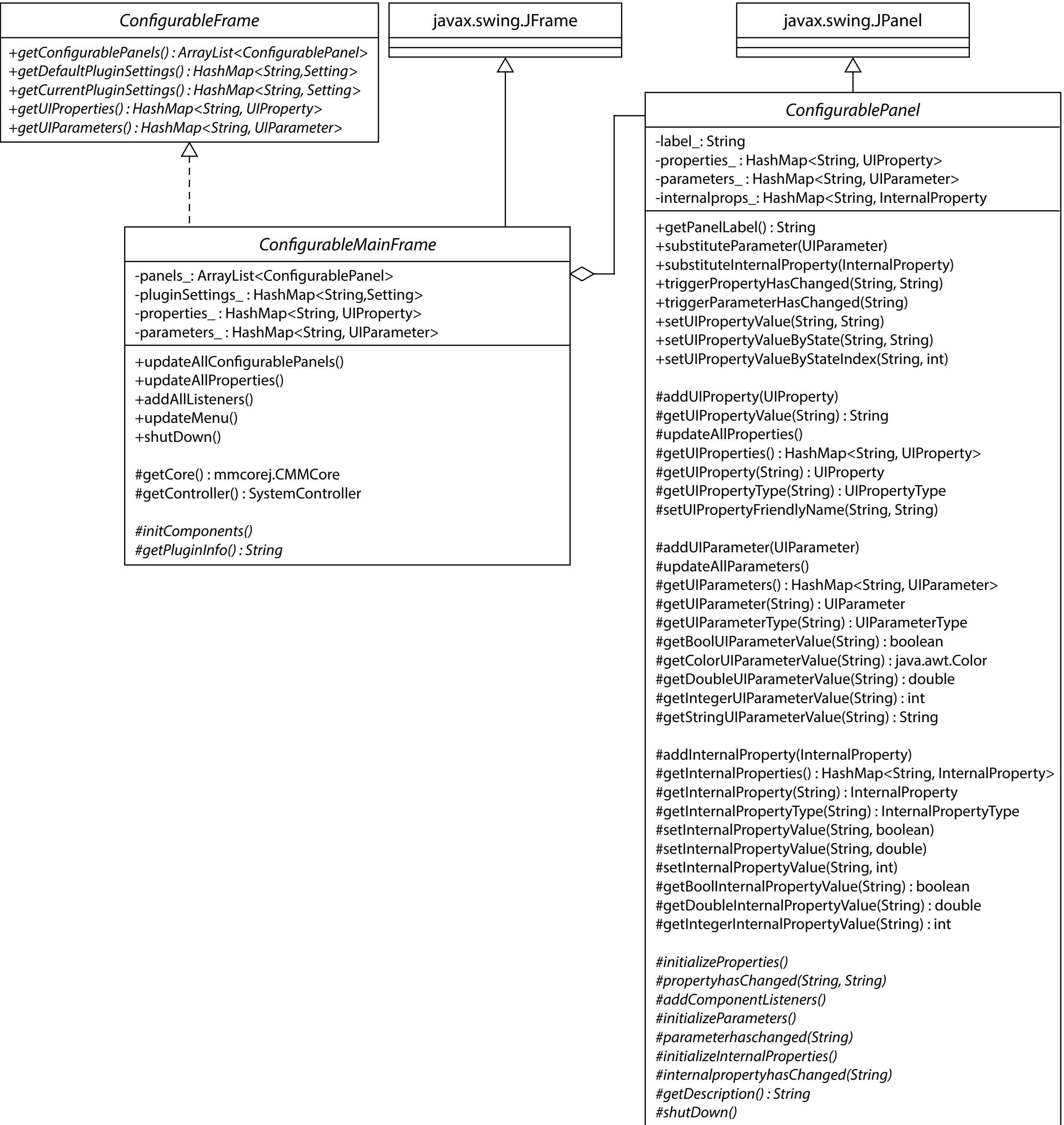

Supplement: Supplementary file 1 — Additional file 1 UML diagram: ConfigurableMainFrame and ConfigurablePanel. Unified Modeling Language (UML) diagram of the ConfigurableMainFrame and ConfigurablePanel classes. The two abstract classes inherit from the Swing classes JFrame and JPanel, respectively. Additionally, ConfigurableMainFrame implements the ConfigurableFrame interface. As both classes are abstract, their abstract methods must be implemented by developers when creating the ConfigurableMainFrame subclass and ConfigurablePanel subclasses. Each class is represented by a box with three compartments: class name, class variables and class methods. Abstract class and interface names are written in italic. Inheritance relationship between two classes is shown as a solid line with an arrow pointing towards the superclass, while implementation of an interface is shown as a dashed line with an arrowhead pointing to the interface class. Aggregation, or class instances being owned by another class, is shown as a solid line with diamond head. The visibility of member variables and methods is indicated by the following signs: - (private), # (protected) or + (public). The diagram shows all private member variables as well as all non-private member methods. Abstract methods are displayed in italic. Variable and method return types, if applicable, are indicated after a colon. The corresponding Javadoc is available in the EMU source-code [25]. [file 12859_2020_3727_MOESM1_ESM.pdf]

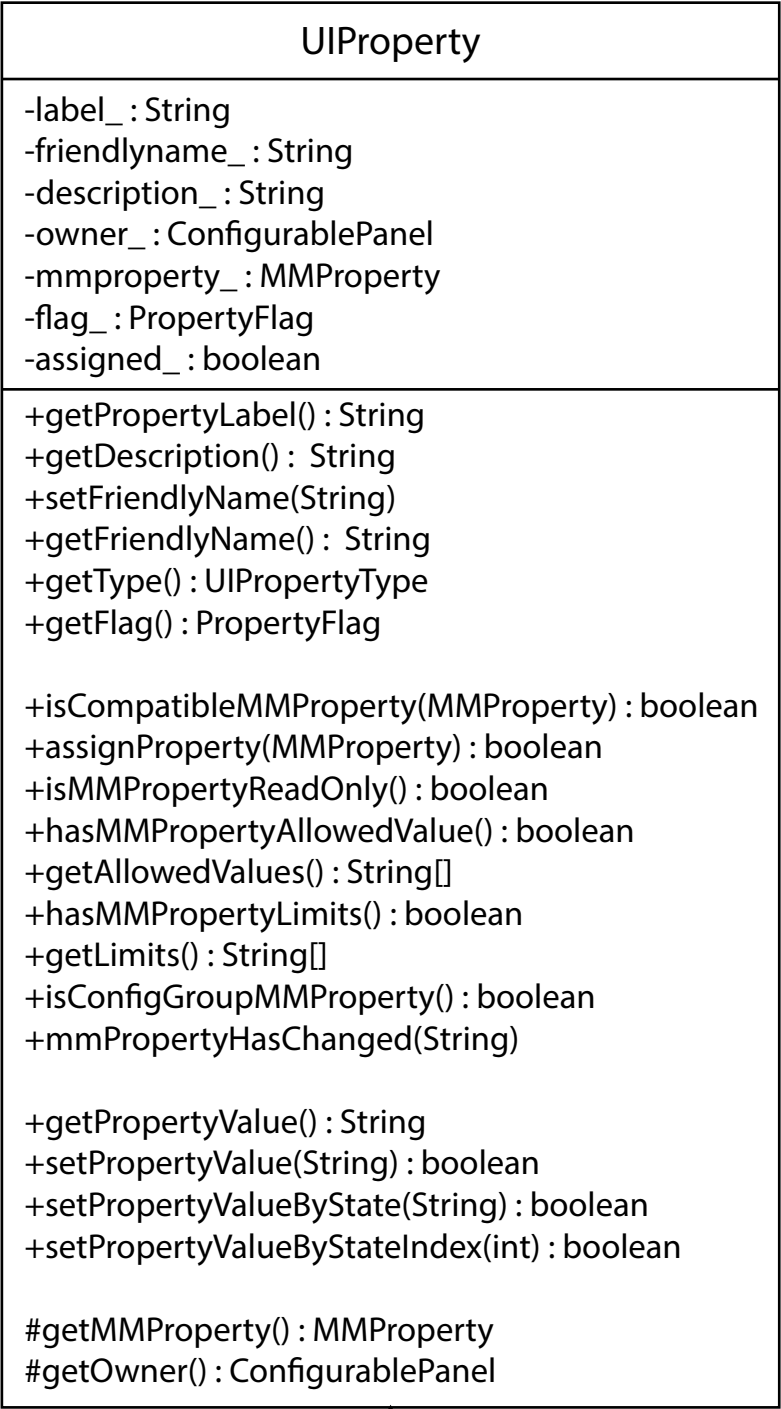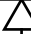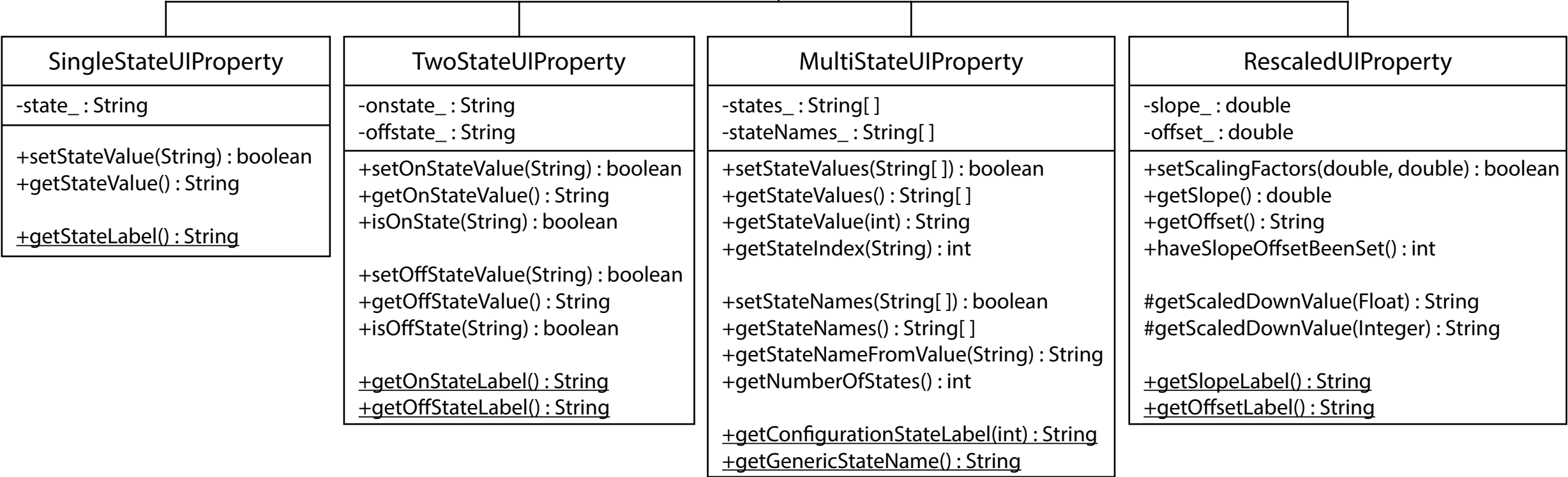

Supplement: Supplementary file 2 — Additional file 2 UML diagram: UIProperty and subclasses. UML diagram of the UIProperty class and its subclasses. These classes are instantiated in ConfigurablePanel subclasses and are ultimately mapped using the EMU configuration to µManager device properties. Descriptions of each class’ specificity can be found in Table 1. Each class is represented by a box with three compartments: class name, class variables and class methods. Inheritance relationship between two classes is shown as a solid line with an arrow pointing towards the superclass. The visibility of member variables and methods is indicated by the following signs: - (private), # (protected) or + (public). The diagram shows all private member variables as well as all non-private member methods. Static methods are underlined. Variable and method return types, if applicable, are indicated after a colon. The corresponding Javadoc is available in the EMU source-code [25]. [file 12859_2020_3727_MOESM2_ESM.pdf]

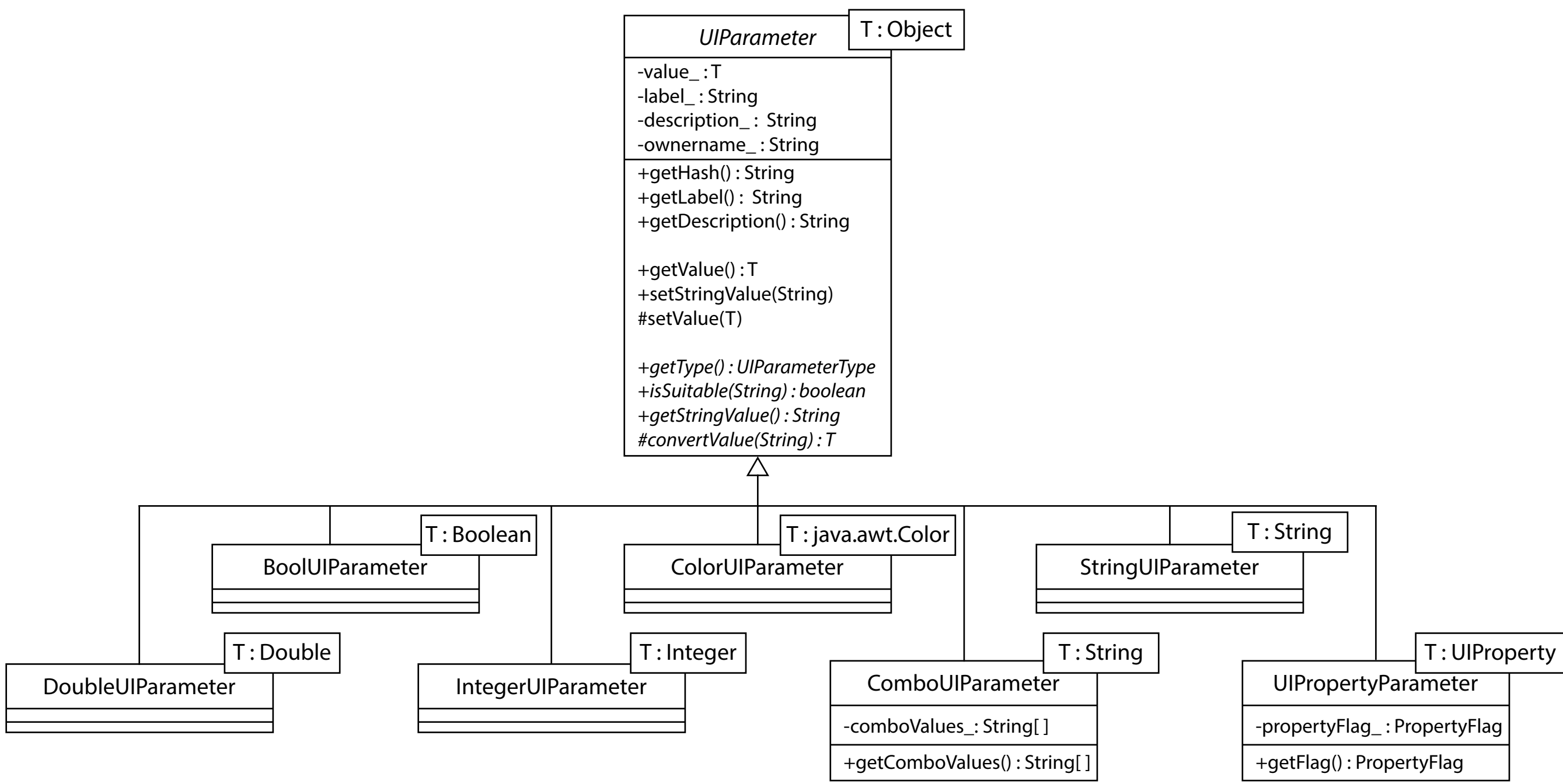

Supplement: Supplementary file 3 — Additional file 3 UML diagram: UIParameter and subclasses. UML diagram of the abstract UIParameter class and its subclasses. These classes are instantiated in ConfigurablePanel subclasses and their respective member variable value_ is set to a user-defined value using the EMU configuration. Each class is represented by a box with three compartments: class name, class variables and class methods. Abstract class names are written in italic. Parameterized class types are shown in a box on the top right corner of each class. Inheritance relationship between two classes is shown as a solid line with an arrow pointing towards the superclass. The visibility of member variables and methods is indicated by the following signs: - (private), # (protected) or + (public). The diagram shows all private member variables as well as all non-private member methods. Abstract methods are displayed in italic. Variable and method return types, if applicable, are indicated after a colon. The corresponding Javadoc is available in the EMU source-code [25]. [file 12859_2020_3727_MOESM3_ESM.pdf]

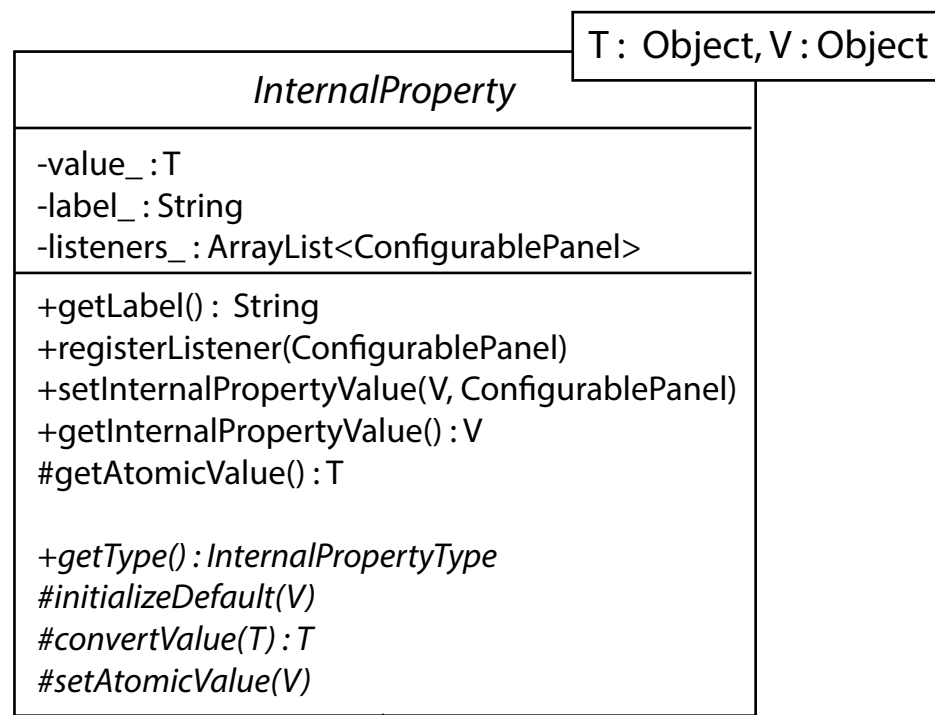

BoolInternalProperty

DoubleInternalProperty

IntegerInternalProperty

Supplement: Supplementary file 4 — Additional file 4 UML diagram: InternalProperty and subclasses. UML diagram of the abstract InternalProperty class and its subclasses. Internal properties are shared between ConfigurablePanel subclasses, provided that they are of the same type and are given the same label. Each class is represented by a box with three compartments: class name, class variables and class methods. Abstract class names are written in italic. Parameterized class types are shown in a box on the top right corner of each class. Inheritance relationship between two classes is shown as a solid line with an arrow pointing towards the superclass. The visibility of member variables and methods is indicated by the following signs: - (private), # (protected) or + (public). The diagram shows all private member variables as well as all non-private member methods. Abstract methods are displayed in italic. Variable and method return types, if applicable, are indicated after a colon. The corresponding Javadoc is available in the EMU source-code [25]. [file 12859_2020_3727_MOESM4_ESM.pdf]

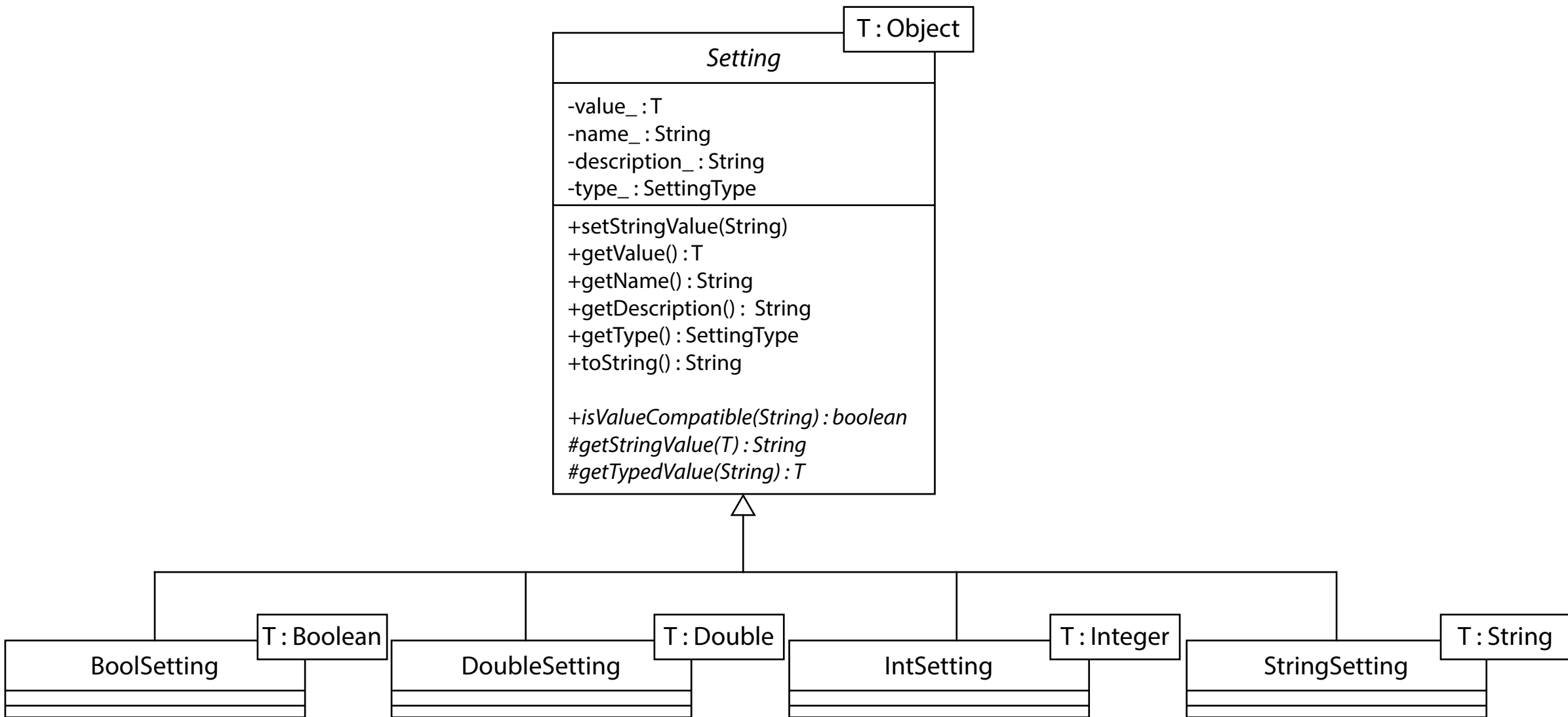

Supplement: Supplementary file 5 — Additional file 5 UML diagram: Setting and subclasses. UML diagram of the abstract Setting class and its subclasses. These classes are instantiated in the ConfigurableMainFrame subclass and their respective member variable value_ is set to a user-defined value using the EMU configuration. Each class is represented by a box with three compartments: class name, class variables and class methods. Abstract class names are written in italic. Parameterized class types are shown in a box on the top right corner of each class. Inheritance relationship between two classes is shown as a solid line with an arrow pointing towards the superclass. The visibility of member variables and methods is indicated by the following signs: - (private), # (protected) or + (public). The diagram shows all private member variables as well as all non-private member methods. Abstract methods are displayed in italic. Variable and method return types, if applicable, are indicated after a colon. The corresponding Javadoc is available in the EMU source-code [25]. [file 12859_2020_3727_MOESM5_ESM.pdf]

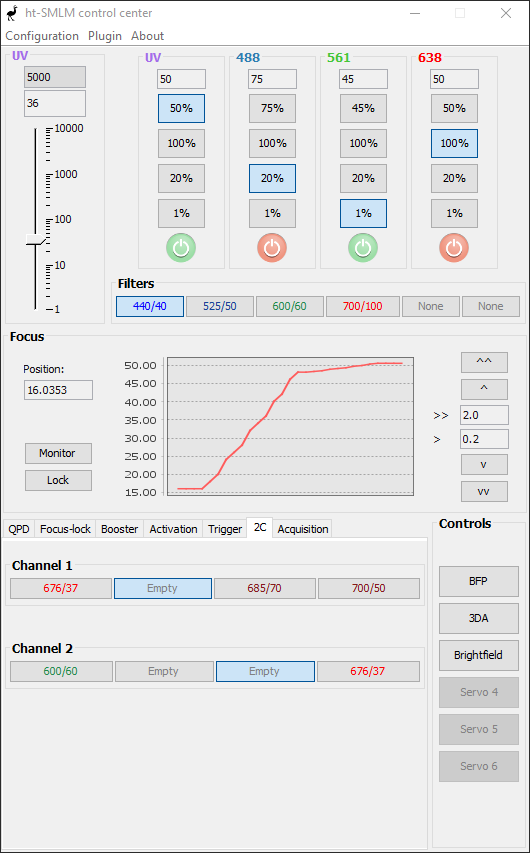

Supplement: Supplementary file 6 — Additional file 6 htSMLM plugin. htSMLM is a complex EMU plugin aimed at controlling a wide-field or localization microscope. Besides the controls for multiple lasers, filter wheels and focus, it features tools to perform series of sequential acquisition (e.g. localization microscopy, multi-slice localization, time series or z-stack) and automated laser activation. [file 12859_2020_3727_MOESM6_ESM.png]
